# The first gapless, reference-quality, fully annotated genome from a Southern Han Chinese individual

**DOI:** 10.1093/g3journal/jkac321

**Published:** 2023-01-11

**Authors:** Kuan-Hao Chao, Aleksey V Zimin, Mihaela Pertea, Steven L Salzberg

**Affiliations:** Department of Computer Science, Johns Hopkins University, Baltimore, MD 21218, USA; Center for Computational Biology, Johns Hopkins University, Baltimore, MD 21218, USA; Center for Computational Biology, Johns Hopkins University, Baltimore, MD 21218, USA; Department of Biomedical Engineering, Johns Hopkins University, Baltimore, MD 21218, USA; Center for Computational Biology, Johns Hopkins University, Baltimore, MD 21218, USA; Department of Biomedical Engineering, Johns Hopkins University, Baltimore, MD 21218, USA; Department of Computer Science, Johns Hopkins University, Baltimore, MD 21218, USA; Center for Computational Biology, Johns Hopkins University, Baltimore, MD 21218, USA; Department of Biomedical Engineering, Johns Hopkins University, Baltimore, MD 21218, USA; Department of Biostatistics, Johns Hopkins University, Baltimore, MD 21211, USA

**Keywords:** genome assembly, annotation, DNA sequencing, reference genome, variant calling

## Abstract

We used long-read DNA sequencing to assemble the genome of a Southern Han Chinese male. We organized the sequence into chromosomes and filled in gaps using the recently completed T2T-CHM13 genome as a guide, yielding a gap-free genome, Han1, containing 3,099,707,698 bases. Using the T2T-CHM13 annotation as a reference, we mapped all genes onto the Han1 genome and identified additional gene copies, generating a total of 60,708 putative genes, of which 20,003 are protein-coding. A comprehensive comparison between the genes revealed that 235 protein-coding genes were substantially different between the individuals, with frameshifts or truncations affecting the protein-coding sequence. Most of these were heterozygous variants in which one gene copy was unaffected. This represents the first gene-level comparison between two finished, annotated individual human genomes.

## Introduction

Over the past 20 years, the biomedical research community has relied on a single human reference genome, which was first published in 2001 (International Human Genome Sequencing Consortium 2001; Venter *et al.* 2001) when it was less than 90% complete, and which has steadily improved since then. That reference genome, currently version GRCh38, is a mosaic of many individuals, which means that it does not capture the actual genome of any single human. In addition, the GRCh38 genome has hundreds of gaps, including very large gaps for all of the centromeres and the short arms of acrocentric chromosomes. In 2022, a breakthrough publication from the Telomere-to-Telomere (T2T) consortium described the first-ever complete human genome, T2T-CHM13, in which all gaps had been filled and which represented a single individual of northern European descent (Nurk *et al.* 2022). Although CHM13 describes a female and is missing the Y chromosome, the T2T consortium finished a gap-free Y chromosome from another individual, HG002, and released that as part of the T2T-CHM13 assembly.

The existence of a finished human genome, together with highly accurate long-read sequencing technology, now allows the assembly of additional human genomes of similarly high quality and completeness. As demonstrated first by the assembly of an Ashkenazy individual, Ash1 (Shumate *et al.* 2020), and more recently by the assembly of a Puerto Rican individual, PR1 (Zimin *et al.* 2021), long-read data (either on its own or in combination with short reads) can be used to generate highly accurate contigs covering most of the human genome, and these can then be scaffolded using either GRCh38 or T2T-CHM13 as a guide. Following scaffolding, the CHM13 sequence can then be used to fill in all gaps, including the centromeres, to produce a gap-free assembly.

An individual genome is far more useful as a research resource if it is annotated. Several computational pipelines can produce annotation *de novo* for a newly assembled genome, but when the genome represents another individual human, as opposed to a new species, comprehensive annotation is not usually necessary. Instead, one can simply map (or “lift over”) the annotation from a reference human genome onto the new assembly, which is usually a superior approach when the reference is of high quality and has reliable annotations. This is the approach we followed.

The first genome sequenced from a Han Chinese individual (designated YH), which was also the first Asian individual genome assembled, was published over a decade ago (Wang *et al.* 2008). Subsequently, another Southern Han (SH) Chinese individual genome, HX1, was *de novo* assembled using single-molecule real-time long reads (Shi *et al.* 2016). Research then shifted toward northern individuals: the first Northern Han (NH) Chinese individual genome, NH1.0, appeared in 2019 (Du *et al.* 2019), and the most recent Han Chinese haplotype-resolved genome, HJ-H1 and HJ-H2, was released in early 2022 (Yang *et al.* 2022). In addition to these four Han Chinese genomes, two other individual genomes, each representing an ethnic group closely related to Han, have been assembled: ZF1 (a Tibetan individual) (Yang *et al.* 2022) and TJ1.p0/TJ1.p1 (a Tujia individual) (Lou *et al.* 2022). However, all these genomes still contain gaps and none were fully annotated. Table 1 provides a comparison among these genomes and the new Han1 assembly.

**Table 1.** A comparison among Han1, GRCh38, and the assemblies of previously released Chinese genomes.

Genome	Ethnicity	Contig N50 (Mb)	Number of contigs	Number of gaps	Assembly size (Gb)
Han1^a^	SH Chinese	148.02	25	0	3.10
HG00621 (hifiasm)^b^	SH Chinese	95.77	182	157	3.11
T2T-CHM13v2.0^c^	Northern European	150.62	25	0	3.12
HJ-H1^d^	NH Chinese	28.15	1,330	427	3.07
HJ-H2^d^	NH Chinese	25.90	896	390	2.91
NH1^d^	NH Chinese	3.60	11,019	8,484	2.89
HX1^d^	SH Chinese	8.33	5,843	4,025	2.93
YH2.0^e^	SH Chinese	0.02	361,157	235,514	2.91
TJ1.p0^f^	Tujia	13.67	1,430	907	2.87
TJ1.p1^f^	Tujia	13.70	1,426	873	2.87
ZF1^g^	Tibetan	23.62	1,384	1360	2.85
GRCh38.p14^h^	Mixed	57.88	994	804	3.10

Assembly sizes include estimated gaps.

^a^Statistics based on chromosomes 1–22, X, Y and mitochondrial DNA of Han1 (CHM13 reference-guided assembly).

^b^Statistics based on primary contigs of HG00621 hifiasm *de novo* assembly.

^c^Statistics calculated directly from the T2T-CHM13 assembly, v2.0.

^d^Statistics taken from Table 1 and results of Yang *et al.* (2022).

^e^Statistics calculated directly from the YH2.0 assembly, downloaded from NCBI at https://www.ncbi.nlm.nih.gov/assembly/GCA˙000004845.2/.

^f^Statistics taken from Table 1 and Supplementary Table S2 of Lou *et al.* (2022).

^g^Statistics calculated directly from the ZF1 assembly, downloaded from the ZF1 Genome Sequence Archive page at https://ngdc.cncb.ac.cn/bioproject/browse/PRJCA000936.

^h^Statistics calculated directly from the GRCh38 assembly, downloaded from NCBI at https://www.ncbi.nlm.nih.gov/assembly/GCF˙000001405.26/. Statistics calculated using the primary assembly only, excluding alternative scaffolds. Scaffolds were split into contigs at position where the number of Ns was greater than 10.

Here we describe the assembly of deep-coverage long-read data to produce the genome of a Southern Han Chinese male individual, which we designate Han1. We filled in all gaps using CHM13 sequence where necessary, and then annotated the genome by mapping over the latest RefSeq annotation from T2T-CHM13 onto Han1. We then compare the annotation between Han1 and T2T-CHM13 and report on some of the key differences.

## Materials and methods

### Sample background

The data for the HG00621 genome were generated and made publicly available by the Human Pangenome Reference Consortium (https://github.com/human-pangenomics/hpgp-data). HG00621 is a Southern Han Chinese male from Fu Jian Province located on the southeastern coast of China. We chose HG00621 for this study because it represents a population for which no annotated genome previously existed, and because Han is the largest ethnic group in the world. The estimated population size of Han people is approximately 1.4 billion, with around 1.29 billion living in China (National Bureau of Statistics of China 2021) and 21.8 million living in Taiwan (Central Intelligence Agency 2022). Moreover, as the largest population is located in East Asia, the Han group plays a key role in understanding ancient human migrations, since it is on the path connecting Africa to the Pacific Islands (Cavalli-Sforza 1998; Zhang *et al.* 2007).

Han Chinese are divided into two population groups, the NH and SH, a division strongly supported by genetic markers as well as dental, craniometric, and archaeological evidence (Tongmao *et al.* 1987; Zhao and Dao Lee 1989; Cavalli-Sforza 1998; Chu *et al.* 1998). One of the reasons that we chose a SH Chinese individual is because most Han immigrants to the United States of America, Taiwan, Singapore, Malaysia, Philippines, and other territories originated from southern China (Cavalli-Sforza 1998). A complete and accurate Han Chinese reference genome should be a valuable resource for genetic studies in this population. Another benefit of selecting HG00621 is that sequencing data are available from both of his parents, HG00619 and HG00620, which provides opportunities to study allelic variations in the future. Also it is worth noting that at least three of HG00621’s grandparents are confirmed SH Chinese individuals.

### Genome assembly

Our main goal in this study was to create a gapless, reference-quality, haplotype-merged assembly of the HG00621 genome with 25 sequences representing chromosomes 1 to 22, X, Y, and the mitochondrial DNA. We assembled contig sequences *de novo*, and we used the T2T-CHM13 genome recently published by the T2T consortium to guide the scaffolding process. The T2T-CHM13 genome is far more complete and accurate than the previous standard, GRCh38 (Nurk *et al.* 2022). CHM13 represents a northern European individual, and because it is a female sample, it lacks a Y chromosome. We used version 1.1 of the T2T-CHM13 assembly (https://github.com/marbl/CHM13), a gap-free version augmented with the complete Y chromosome from HG002, an individual of Ashkenazi Jewish descent.

The initial Han1 assembly was created using PacBio high-fidelity (HiFi) reads and Oxford Nanopore Technology (ONT) reads, with approximately 39x coverage in HiFi reads and 35x coverage in ONT reads (Table 2). The basecallers were ccs v4.0.0 (HiFi) and Guppy v4.0.11 (ONT). We assembled all HiFi reads with Hifiasm (Cheng *et al.* 2021) version 0.16.1-r375 and all ONT reads with Flye (Kolmogorov *et al.* 2019) version 2.5. The statistics of these two preliminary assemblies are shown in Table 3. Comparing both assemblies, the HiFi draft assembly had substantially higher contiguity with an N50 contig size of 95.8 Mb and only 182 contigs, while the Flye assembly had an N50 of 40.9 Mb and 1,658 contigs. We, therefore, chose the Hifiasm output as the main assembly for the subsequent steps.

**Table 2.** Sequencing data from HG00621 used for assembly of the Han1 genome.

Sequence type	Basecaller	Number of reads	N50 read length (bp)	Mean read length (bp)	Maximum length	Total length (Gbp)	Genome coverage
PacBio HiFi	ccs v4.0.0	5,570,675	21,499	21,989	50,388	122.50	39.45x
ONT Ultralong	Guppy v4.0.11	3,110,293 (9.11% > 100 Kb)	83,822	34,136	2,495,296	106.17	34.75x

**Table 3.** Statistics for the preliminary Han1 assemblies.

Assembler	Sequencing data	assembled sequence (bp)	Contig N50	Number of contigs	Quality value
Hifiasm v0.16.1-r375	PacBio HiFi	3,110,501,483	95,769,069	182	57.8^a^
Flye v2.5	ONT Ultralong	2,974,205,132	40,850,737	1,658	25.6^a^

^a^Quality values were calculated using JASPER v1.0.0.

For the scaffolding step, we first used the MaSuRCA chromosome scaffolder module (Zimin *et al.* 2017) (chromosome_scaffolder.sh) with the T2T-CHM13 reference to order and orient contigs generated by Hifiasm. This process is similar to the scaffolding procedures we used in building the Ashkenazi (Ash1) (Shumate *et al.* 2020) and Puerto Rican (PR1) (Zimin *et al.* 2021) reference genomes. The chromosome scaffolder identified 12 misassembled contigs that we had to split before scaffolding. A list of alignments of these 12 contigs to T2T-CHM13 appears in Supplementary Table S1.

After scaffolding, we had 25 scaffolds (with gaps) representing nuclear chromosomes 1–22, X and Y, plus 77 unplaced contigs containing an additional 38,815,834 bp. The chromosome sequences contained only 101 gaps.

We then proceeded with three gap closing steps. For these steps, we used MaSuRCA’s intra-scaffold gap-closer script (close_scaffold_gaps.sh), which is a wrapper for the SAMBA scaffolder (Zimin and Salzberg 2022). In the first step, we used HiFi reads and default parameters, which closed 24 gaps. The second step used contigs generated by Flye from the ONT reads with modified parameters “-m 2500 -o 10000,” which set the thresholds for the minimum match length on both sides of a gap (2,500 bp) and the maximum overhang (10,000 bp). This step closed an additional 19 gaps. The third and final step of automated gap closing used the CHM13 sequence to attempt closing the remaining 58 gaps. This step closed another 45 gaps with only 7 gaps remaining in chromosomes 13, 14, 15, and 22. Manual inspection of these gaps identified repeat-induced misassemblies in pericentromeric regions in chromosomes 13 and 15, which we corrected manually, and redundant haplotype-variant contigs in chromosomes 14 and 22 that we deleted. A more detailed strategy for solving misassembled contigs is shown in Supplementary Figure S1 and Supplementary Materials, and we listed their positions on Han1 draft assembly in Supplementary Table S2.

After fixing the misassemblies and eliminating redundant haplotype contigs, we re-ran gap closing and ended up with a single contig for every chromosome. We observed that one of the unplaced contigs contained several copies of the mitochondrial sequence, and we extracted a single circular copy using alignment to the mitochondrial sequence from GRCh38.

We screened all 77 unplaced contigs for contamination by using KrakenUniq (Breitwieser *et al.* 2018) to align them to a database containing bacteria, viruses, and common vectors. We identified one contig as the Epstein-Barr virus (EBV), a common DNA viral contaminant in human DNA. The size of this contig was 177,625 bp, which is close to the 170 Kb genome size of EBV (Chang *et al.* 2009). We checked the portal and confirmed that the cell line for HG00621 was immortalized using EBV. This contig was removed. We aligned all remaining unplaced contigs to the nuclear chromosomes and found that all were redundant copies of variant haplotypes.

The last step of the process was to polish the chromosome sequences with the HiFi reads using JASPER (Guo *et al.* 2022) version 1.0.0. JASPER capitalized bases supported by HiFi reads in patches of sequence that were originally filled in using T2T-CHM13, where the DNA sequence was shown in lowercase. This step also produced a quality value (QV) estimate of 57 for the polished assembly, which corresponds to fewer than 2 errors per megabase.

We used three programs in the MUMmer4 package (Marçais *et al.* 2018) to compare the Han1 and T2T-CHM13 genomes: we aligned Han1 to T2T-CHM13 using nucmer, selected the best 1-to-1 alignment using delta-filter, and created dotplots using mummerplot.

### Gene annotation

We used Liftoff (Shumate and Salzberg 2021) version 1.6.2 to map genes from T2T-CHM13 onto Han1. The T2T-CHM13 annotation was generated by mapping RefSeq (O’Leary *et al.* 2016) release 110 of GRCh38.p14 onto T2T-CHM13 version 2.0. The T2T-CHM13 annotation contains a total of 61,140 genes, including 20,022 protein-coding genes and 18,389 lncRNA genes. It includes 181,713 transcripts, of which 129,878 are protein-coding. The T2T-CHM13 annotation used here is available at ftp://ftp.ccb.jhu.edu/pub/data/T2T-CHM13/.

Although Liftoff maps most genes automatically, the ribosomal DNA (rDNA) regions present a problem because they occur in very long tandem arrays of near-identical copies. Each rDNA unit is composed of three ribosomal RNA (rRNA) genes, 18S, 5.8S, and 28S, separated by two flanking spacers ($5^{\prime }$- and $3^{\prime }$-ETS) and two internal transcribed spacers (ITS-1 and 2) (Agrawal and Ganley 2018). The arrays are located on the acrocentric chromosomes 13, 14, 15, 21, and 22. To accurately map the rDNA arrays onto Han1, we adopted a two-pass Liftoff process.

First, we ran Liftoff to map all genes except the rDNA arrays onto Han1, as follows. We aligned a 44,838 bp reference rDNA sequence from the KY962518 locus (Kim *et al.* 2018) to Han1 using Nucmer (Marçais *et al.* 2018) with the parameters: “--maxmatch -l 31 -c 100,” which identified 1,196 preliminary rDNA locations. We then filtered these matches to retain only those that were >1,000 bp and >98.5% identical to the reference rDNA sequence, which yielded 257 approximate rDNA array locations. We used bedtools (Quinlan and Hall 2010) maskfasta to mask the locations of the 257 potential rDNA arrays, replacing them with Ns, and then ran Liftoff with parameters: “-chroms chroms_mapping.csv -copies -polish” to map all CHM13 genes except the rDNA arrays. This Liftoff first-pass procedure mapped 60,099 genes, including 20,003 protein-coding genes, 18,321 lncRNAs, and 51 non-rDNA array rRNAs, as well as many other gene types including microRNAs and pseudogenes.

In our second-pass process, we only lifted over the rDNA annotations from T2T-CHM13 onto Han1. We first extracted all rDNA annotations on T2T-CHM13 into an annotation (GFF) file, which included 219 rDNA units with three subunits each, and thus 657 subunits in total. Then we ran Liftoff with parameters: “-f features.txt -mm2_options ‘-N 250’ -copies -sc 0.95.” The features.txt restricted the mapping to locations where all three subunits mapped onto Han1 at the same locus in the correct order, 18S, 5.8S, and 28S, which we called a valid rDNA unit lift-over condition. One additional note is that the rDNA morphotypes on each chromosome are structurally distinct (Nurk *et al.* 2022) which can create a mapping bias. We checked all 219 rDNA arrays during the mapping process to ensure that each copy was mapped to the best-matching chromosome. The Liftoff second-pass procedure mapped a total of 203 rDNA units, which is 609 subunits in total.

To verify the rDNA mapping result, we intersected the mapped annotations from the first and second passes using bedtools (Quinlan and Hall 2010) intersect version 2.30.0 with parameters: “-wa -wb.” None of the 203 rDNA units overlap with any other genes mapped to Han1, which shows no sign of mismapped rDNA units. In the final step, we merged the two GFF files and ran gffread (Pertea and Pertea 2020) version 0.12.7 with parameters: “-O -F --keep-exon-attrs” to sort the final annotation and set the phases of CDS features.

### T2T-CHM13 and Han1 annotation comparison

We compared the gene annotations between T2T-CHM13 and Han1 by running LiftoffTools (Shumate and Salzberg 2022) version v0.2.0, which performed a transcript-level comparison between all transcripts on the two genomes. For protein-coding transcripts, LiftoffTools determines the effects of any DNA differences on the protein sequence, which it categorizes into 10 groups: synonymous, nonsynonymous, in-frame deletion, in-frame insertion, start codon loss, $5^{\prime }$ truncation, $3^{\prime }$ truncation, frameshift, and stop codon gain.

Liftofftools reported comparisons between 181,029 transcripts between T2T-CHM13 and Han1, with only 720 transcripts unmapped. For 130,909 transcripts with CDS features (which included protein-coding genes, VDJ segments, and some pseudogenes), this step computed DNA sequence identity, amino acid sequence identity, and any of the 10 variant effects aforementioned. For the 50,120 transcripts lacking CDS features, only the DNA sequence identity was computed.

To analyze mutations at the gene level, we created a transcript-gene look-up table by their IDs, mapped 130,909 transcripts back to their gene names and obtained a list of 19,706 protein-coding genes, 25 C regions, 75 J segments, and 403 V segments, for a total of 20,209 genes. For each of these, we selected the transcript with the highest amino acid sequence identity score as representative and categorized the gene into the variant effect group of that transcript. The number of genes in each group is summarized in Table 4.

**Table 4.** Comparison of genes with CDS features mapped from T2T-CHM13 to Han1.

Mapped genes with CDS features								
Conserved genes (82%)		Altered genes (18%)						
Identical	Synonymous	Nonsynonymous	Start lost	Stop gained	Truncated	Frameshift	In-frame insertion	In-frame deletion
14,130	2,442	3,200	21	31	1+39 ($5^{\prime }+3^{\prime }$)	143	110	92

For this gene-level analysis, the transcript-to-transcript mapping selected the alignment that produced the highest amino acid identity score for the coding sequence (CDS). Truncated genes are those where the mapped copy is missing either the $5^{\prime }$ end or the $3^{\prime }$ end of the transcript, including the start/stop codon.

We were particularly interested in genes containing deleterious mutations. We assumed that nonsynonymous mutations, in-frame insertions, and in-frame deletions are relatively harmless (Dunkle and Dunham 2015), and we focused on mutations that created major differences between the T2T-CHM13 and Han1 protein sequences, which included frameshifts (an insertion or deletion whose length is not a multiple of 3), truncations that removed either the $5^{\prime }$ or $3^{\prime }$ end of the transcript, including part of the CDS, mutations that changed the start codon to another codon, and mutations that created a premature stop codon. This gave us a target list of 235 genes for further investigation.

Because many of the 235 genes with deleterious mutations might represent cases where the Han1 genome is heterozygous, and where one haplotype contains a normal copy of the gene, we went back to the raw HiFi data and used it to identify heterozygous and homozygous variants, as follows. We first aligned all HiFi reads to the T2T-CHM13 genome using Minimap2 (Li 2018) version 2.24-r1122, and then used SAMtools (Li *et al.* 2009) version 1.13 to sort and index the aligned BAM file. We called variants using the “mpileup” and “call” commands from BCFtools (Li 2011) version 1.15.1 and selected heterozygous and homozygous variants by running “bcftools filter” with parameters “GT=‘het’” and “GT=‘hom’,” respectively. This step identified 4,331,520 heterozygous sites and 2,285,451 homozygous sites.

We manually inspected a sample of altered genes with heterozygous mutations and found in each case that the alternative haplotype lacked the deleterious mutation. Therefore, we focused the remainder of our analysis on genes with homozygous mutations. We filtered out single nucleotide polymorphisms (SNPs) and kept mutations that might contribute to frameshifts, leaving us with 204,706 homozygous mutation sites.

Next, we extracted all CDS regions from all transcripts of the 235 genes with the most deleterious mutations, which yielded a total of 8,578 coding exons. We intersected these with the 204,706 homozygous, non-SNP mutation sites using bedtools intersect. After removing repeats caused by exon sharing between isoforms, 54 distinct mutation sites in 46 distinct genes were identified, where each gene contained at least 1 homozygous, non-SNP mutation as compared to T2T-CHM13.

### Protein-level analysis of altered genes

We further investigated our list of 46 altered genes. We first checked the reported homozygous mutation sites in IGV (Robinson *et al.* 2011), extracted the GFF entries with target gene names, and translated their CDS into amino acid sequences using gffread (Pertea and Pertea 2020). We then compared the protein sequence of Han1 and T2T-CHM13 to RefSeq using BLASTP (Altschul *et al.* 1997) version 2.12.0+ and parasail (Daily 2016) version 1.2.1 to align the sequences and calculate the amino acid sequence identity.

### Gene copy number analysis

We compared copy number variation between the Han1 and T2T-CHM13 by grouping genes into paralogous groups using Liftofftools, which uses the “linclust” function in MMSeq2 (Steinegger and Söding 2017) version 13.45111. Protein-coding genes were clustered based on their amino acid sequences, and non-coding genes were clustered based on their nucleotide sequences, where genes in a cluster needed to be at least 90% identical over 90% of their lengths to at least one other cluster member.

## Results and discussion

### Genome assembly

**Fig. 1.**
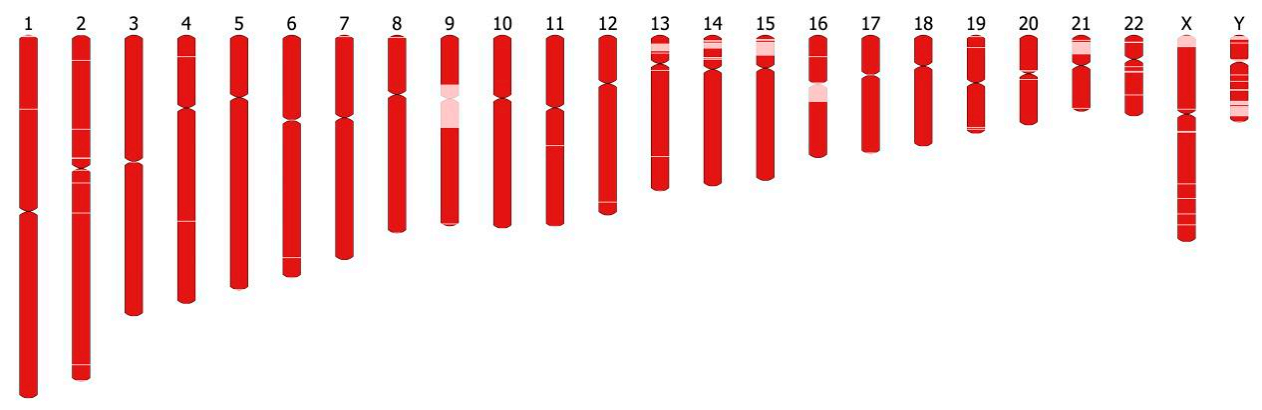
A visualization of the 24 chromosomes in Han1. Regions in red were assembled from HG00621, and regions in light pink are sequences inserted from CHM13. Graphics were created using Ideogram.js version 1.37.0 (https://github.com/eweitz/ideogram).

The Han1 genome (version 1.0) contains 3,099,707,698 bp, and the autosomes (1–22), the sex chromosomes (X and Y), and the mitochondrial genome were assembled end-to-end with no gaps. Approximately 120 Mb in gaps was filled using sequence from the T2T-CHM13 genome, illustrated in Fig. 1. The sizes of each chromosome and the amount of non-HG00621 sequence per chromosome are summarized in Table 5. Han1 is slightly smaller than T2T-CHM13, which has a total size of 3.117 Gbp. Chromosomes 9 and Y have the lowest proportion of HG00621 sequence compared to other chromosomes. Chromosome 9 has the largest centromeric region among all autosomes, as has been previously reported (Humphray *et al.* 2004). The centromeric region spans approximately 35 Mb, which was inserted entirely from T2T-CHM13. Chromosome Y contains a number of particularly challenging regions for assembly, including the male-specific region, MSY, which is composed of eight very long palindromes ranging from 9 Kb (P7) to 1.45 Mb (P1) with arm-to-arm sequence identities of 99.94–99.997% (Rozen *et al.* 2003; Skaletsky *et al.* 2003), as well as a lengthy X-transposed region, XTR, sharing 99% sequence identity to chromosome X (Skaletsky *et al.* 2003; Ross *et al.* 2005), which led us to rely on the T2T-CHM13 assembly for 27% of chromosome Y. Note that the T2T-CHM13 Y chromosome was derived from a separate individual, HG002, of Ashkenazi Jewish descent.

**Table 5.** Han1 chromosome sizes and the amount of non-HG00621 sequence per chromosome in the final Han1 assembly.

Chromosome	Han1 total (bp)	Non-HG00621 sequence (inserted from CHM13) (bp)	Ratio of source sequences (HG00621:CHM13)
1	249,525,787	119,184	0.9995
2	242,739,747	2,482,037	0.9898
3	200,211,729	377,991	0.9981
4	192,045,028	518,393	0.9973
5	181,667,637	494,129	0.9973
6	170,861,069	314,798	0.9982
7	160,865,769	107,243	0.9993
8	145,880,131	791,768	0.9946
9	148,018,047	35,504,706	0.7601
10	135,316,043	585,347	0.9957
11	135,129,219	874,841	0.9935
12	134,132,185	102,971	0.9992
13	111,903,191	10,782,722	0.9036
14	101,435,482	5,090,291	0.9498
15	101,210,777	12,429,469	0.8772
16	95,412,483	13,280,238	0.8608
17	83,450,189	1,080,955	0.9870
18	78,996,361	210,798	0.9973
19	61,978,944	1,089,081	0.9824
20	65,189,243	963,587	0.9852
21	45,827,290	5,613,897	0.8775
22	50,610,422	5,397,082	0.8934
X	154,227,164	7,056,525	0.9542
Y	53,057,190	14,607,629	0.7247
M	16,571	0	1.0000
Total	3,099,707,698	119,875,682	0.9614

Every chromosome is in a single contig.

We identified two nuclear mitochondrial sequences (NUMTs) (Lopez *et al.* 1994) in Han1. The first one, an 866 bp insertion on chromosome 13 from 12,339,933 to 12,340,799 is present in T2T-CHM13, but absent from GRCh38. The second NUMT is a longer 13,781 bp insertion on chromosome 20 from 21,541,750 to 21,555,531, and it is unique to the Han1 genome, i.e. it is absent from both T2T-CHM13 and GRCh38. We validated these two NUMTs by aligning the ONT reads back to the Han1 genome using minimap2 with the parameter “--secondary=no,” requiring that the reads contained the entire NUMT and that the alignments extended at least 500 bp beyond it on both ends. We found 15 ONT reads supporting the NUMT on chromosome 13, suggesting that it may be a heterozygous insertion, and 26 ONT reads supporting the chromosome 20 NUMT. The chromosome 20 NUMT includes 26 genes, of which 8 are protein-coding genes, 16 are tRNAs, and 2 are rRNAs.

**Fig. 2.**
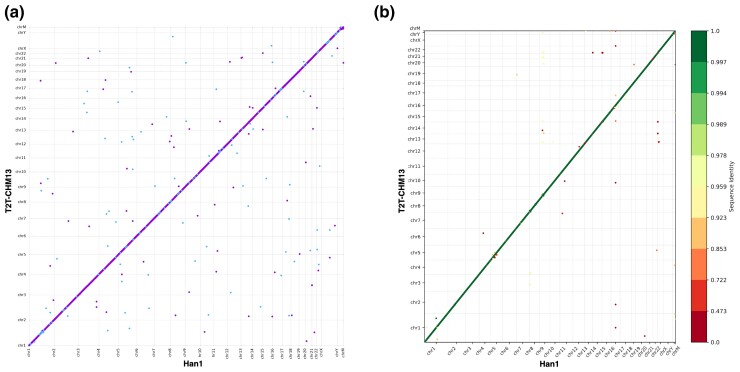
a) A dot plot between the DNA sequences of Han1 and T2T-CHM13 shows the high collinearity of every chromosome between the two genomes. The segments in purple color mean sequences in T2T-CHM13 and Han1 are in the same direction, whereas the blue color means they are in the reverse direction. b) A gene order plot, where genes were numbered from 1 to N in order along all chromosomes of both T2T-CHM13 and Han1, and color-coded according to sequence identity. For both a) and b), the X axis is the T2T-CHM13 reference, and the Y axis is Han1. Chromosomes are in the order of 1 to 22, X, Y, and M. The alignments and dot plot were produced using the MUMmer4 software, and the gene order plot was generated by Liftofftools v0.2.0.

**Fig. 3.**
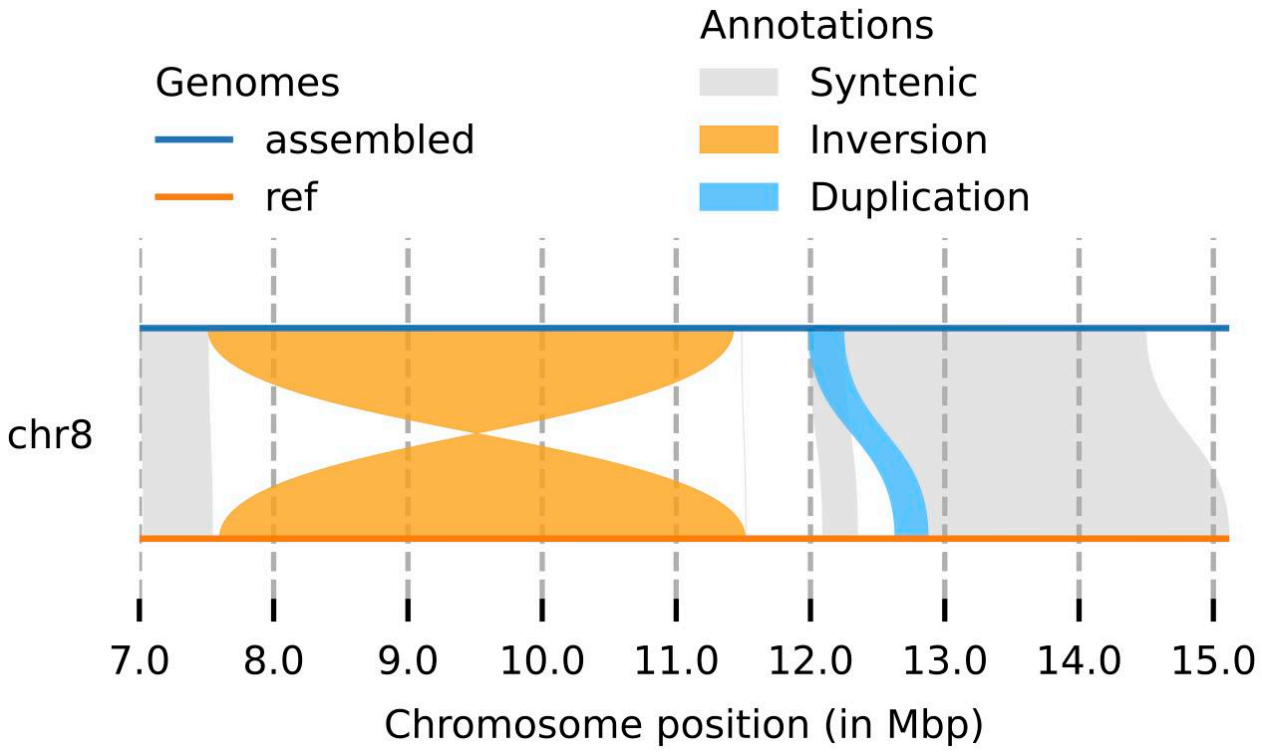
A detailed view of an inversion between Han1 (top, in blue color) and T2T-CHM13 (bottom, in orange color), spanning the region from approximately 7.5 to 11.5 Mbp on chromosome 8. Figure created with Plotsr (Goel and Schneeberger 2022) version 0.5.4.

A dot plot comparing the entire sequence of Han1 and T2T-CHM13 genomes is shown in Fig. 2, which illustrates the overall high collinearity of all chromosomes. One interesting finding is that Han1 contains an inversion at chr8: 7,306,407–11,410,283 (4,103,876 bp) which corresponds to T2T-CHM13 chr8: 11,717,452–7,617,994 (4,099,458 bp). This inversion is one of the largest common inversion polymorphisms in humans (Logsdon *et al.* 2021), and the arrangement in Han1, shown in more detail in Fig. 3, matches GRCh38 but disagrees with T2T-CHM13. A more detailed dotplot of the inversion is shown in Supplementary Figure S2. This region includes the β-defensin gene cluster locus and has been described as one of the most structurally dynamic regions in the human genome (Ganz 2003; Hollox *et al.* 2003, 2008; Mohajeri *et al.* 2016).

### Gene content differences between Han1 and T2T-CHM13

The recent completion of the genome of a single individual, T2T-CHM13, along with the results here for Han1, provided us with the opportunity to do the first-ever direct comparison between the gene content of two complete individual human genomes. Because Han1 was assembled using T2T-CHM13 as a guide, it may have a gene content bias toward T2T-CHM13. In total, the Han1 annotation contains 60,708 genes, including 20,003 protein-coding genes, 18,321 lncRNAs, and 658 rRNAs. The number of genes on Han1 is slightly lower than the 61,140 genes and 20,022 protein-coding genes on T2T-CHM13, although the small differences could be due to mapping artifacts. An overview of the comparison between Han1 and T2T-CHM13 by gene categories, along with a comparison to the gene content of GRCh38, is shown in Table 6.

**Table 6.** A comparison of major gene categories in the annotations of GRCh38.

Gene biotype	Count in GRCh38	Count in T2T-CHM13	Count in Han1
Protein coding	19,871	20,022	20,003
lncRNA	17,793	18,389	18,321
Pseudogene	15,357	16,027	15,881
miRNA	1,914	2,046	2,058
Transcribed pseudogene	1,221	1,262	1,232
rRNA	38	765	658
Other	2,506	2,629	2,556
Total	58,700	61,140	60,708

As described in Materials and methods section, we identified 46 distinct protein-coding genes that appear to have deleterious mutations in both copies of the gene. Among these, 27 are frameshifts, 14 are $3^{\prime }$ truncations, 3 have lost their start codons, and 2 have gained an early stop codon. A list of the genes in each category appears in Supplementary Table S3. Out of these 46 genes, 24 are hypothetical proteins with no known function, raising the question of whether they are actually functional proteins. Six of these are olfactory receptors, which might indicate a genuine difference in the number of olfactory receptors, or might indicate that those copies are pseudogenes. Three are not actual genes but VDJ segments, which are highly diverse among individuals.

The remaining 13 genes are listed in Table 7. Two of these genes, MUC19 and AQP12A, contain premature stop codons and appeared to be severely truncated in Han1, with protein lengths far shorter than the corresponding proteins in both T2T-CHM13 and GRCh38.

**Table 7.** Protein level comparison between the Han1 and T2T-CHM13 genomes, showing genes with homozygous mutations in Han1 that are either frameshifted or truncated with respect to T2T-CHM13.

		Protein length		Protein length ratio (vs RefSeq)		Amino acid identity score (vs RefSeq)	
Gene name	RefSeq protein length	Han1	T2T-CHM13	Han1	T2T-CHM13	Han1	T2T-CHM13
MUC19	6,985	1,241	5,332	0.178	0.763	0.278	0.943
AQP12A	295	163	295	0.553	1.000	0.551	0.986
RETNLB	111	111	14	1.000	0.126	1.00	0.295
TCP11X1	407	407	201	1.000	0.494	0.997	0.417
DEFB126	111	111	82	1.000	0.739	0.936	0.576
TPSB2	275	275	166	1.000	0.604	0.927	0.584
PBOV1	135	136	135	1.010	1.000	1.000	0.875
GOLGA6L10	536	550	522	1.026	0.974	0.945	1.000
KLHDC7B	1,235	1,211	1,215	0.981	0.984	0.978	0.981
NBPF19	3,843	3,283	3,772	0.854	0.982	0.927	0.961
RP1L1	2,400	2,417	2,464	1.007	1.027	0.989	0.970
TMEM82	343	343	344	1.000	1.000	1.000	0.994
KIR2DL3	341	341	341	1.00	1.000	1.000	0.956

The RefSeq protein length is based on the RefSeq v110 annotation of GRCh38.

Five genes, RETNLB, TCP11X1, DEFB126, TPSB2, and PBOV1, have protein sequences that are well-conserved compared to GRCh38, with 93–100% identity. These genes appeared to be frameshifted in T2T-CHM13, creating protein products in T2T-CHM13 that are either much shorter or frameshifted, with amino acid identity ranging from 30% to 88%. These results indicate that the deleterious mutations occur in the T2T-CHM13 genome (which we note was derived from a complete hydatidiform mole, not from a healthy adult) rather than in Han1.

For the other six genes, GOLGA6L10, KLHDC7B, NBPF19, RP1L1, TMEM82, and KIR2DL3, the translations of their CDS regions are well-conserved despite the mutations, with all of their amino acid identity scores higher than 92% and protein length ratios close to one.

### Conserved synteny

Figure 2b shows the overall conserved gene order between the Han1 and T2T-CHM13 genomes. The figure shows 60,746 points, each representing a distinct gene, plotted in their order along the chromosomes. The color coding indicates the sequence identity score between T2T-CHM13 genes and Han1 genes, with green higher and red lower. Among the 60,746 genes, around 98.2% of genes (59,654) have sequence identity scores greater than 95%. As the figure shows, nearly all genes occur in exactly the same order between the two genomes.

### Gene family expansions

Liftofftools clustered all protein sequences using relatively strict criteria, which identified 19,653 clusters of near-identical proteins, of which 122 clusters had at least three genes in T2T-CHM13. In total, 94 of the 19,653 clusters have copy number changes, with all changes being copy number losses in Han1.

We looked more closely at the TSPY gene family, which occurs as a large tandem array with interspersed repeats on chromosome Y in both T2T-CHM13 and Han1. Both genomes have 46 copies of TSPY, and CHM1 has 20 pseudogenes, while Han1 has 24. It is worth noting that one copy of TSPY3 in T2T-CHM13, although annotated as a protein-coding gene, has a premature stop codon that creates a truncated 237 amino acids (aa) protein, whereas the same gene on Han1 is full length at 308 aa. Thus, T2T-CHM13 appears to have lost one copy of the TSPY gene family.

In the 94 clusters with copy number loss, we found 46 clusters in T2T-CHM13 that had no corresponding genes in Han1, 43 clusters that lost a single gene copy, and five clusters that had lost more than one copy. The last groups (clusters that lost >1 gene copy) are shown in Supplementary Table S4.

### Conclusion

In this study, we assembled and annotated the first gap-free genome, Han1, from a SH Chinese individual. The genome has a total size of 3,099,707,698 bp, and the autosomes (1–22), the sex chromosomes (X and Y), and the mitochondrial genome are assembled end-to-end with no gaps. The vast majority of the sequence is collinear with the T2T-CHM13 and GRCh38 genomes, although we did observe at least one novel mitochondrial insertion and a number of small-scale sequence duplications. Annotation of Han1 generated 60,708 putative genes, of which 20,003 are protein-coding genes. Finally, we conducted the first-ever direct comparison between the gene content of two complete individual human genomes, Han1 and T2T-CHM13 to determine how many genes are truly different between individuals. This analysis revealed two protein-coding genes in Han1 and five genes in T2T-CHM13 that appear to be severely truncated in comparison to the full-length version of the protein.

## Supplementary Material

jkac321_Supplementary_Data

## Data Availability

All raw reads for HG00621 were generated and released by the Human Pangenome Reference Center, and are available as an AWS Open Data set from https://github.com/human-pangenomics/hpgp-data. The Han1 assembly (v1.0) is available from GenBank under accession JANJEX000000000 and on Github at https://github.com/JHUCCB/ChineseHanSouthGenome. Supplementary material are available at *G3* online.
