# Supplementary material for: The first gapless, reference-quality, fully annotated genome from a Southern Han Chinese individual
Source: G3 (Bethesda). 2023 Jan 11;13(3):jkac321. doi: 10.1093/g3journal/jkac321 (PMC9997556; doi:10.1093/g3journal/jkac321)
Supplement: jkac321_Supplementary_Data [file jkac321_supplementary_data.pdf]

Supplementary Materials

We used the ‘`chromosome_scaffolder.sh`’ script that is part of MaSuRCA assembler to close gaps in the chromosome scaffolds. `chromosome_scaffolder.sh` is a wrapper for the published SAMBA scaffolder, whose function is to split the scaffolds at gaps (runs of N’s), record which contigs are adjacent in the scaffolds and then run SAMBA to close as many gaps as possible. We first closed gaps using the HiFi reads, then we used the Flye assembly, and finally we used the CHM13 sequence. Some gaps required manual intervention because they could not be closed automatically due to misassemblies in the contigs surrounding the gap. An example of such misassembly is shown in FigureS2. This was the misassembly on chromosome 13 that resulted in a gap that could not be automatically closed due to a misassembly in the contig on the right side of the gap. Another reason for failing to close a gap automatically is presence of redundant haplotype contigs. These contigs would end up next to each other in the chromosome scaffold. We screened for these contigs by aligning them to the bigger contigs surrounding them, and if they aligned with >95% similarity over >75% of their length, we eliminated these contigs. After these fixes we re-ran the chromosome scaffolder to close gaps with the CHM13 sequence and the software was able to close the remaining gaps.

Table S1 MaSuRCA chromosome scaffolder aligned the HG60021 hifiasm assembly back to T2T-CHM13 and identified 12 misassembled contigs. 12 contigs are grouped by colors and listed with their corresponding alignments on T2T-CHM13. They are further split into 30 contigs for the later gap closing step.

Contig	Chromosome	Start position	End position
ptg000002l:1-4002663.3390039	chr21	2237596	2850180
ptg000023l:1-31662945.0	chr22	21259644	37872816
ptg000023l:1-31662945.16613180	chr22	21019534	21259634
ptg000023l:1-31662945.16853284	chr22	18572419	21019533
ptg000023l:1-31662945.19300419	chr22	7043342	18572407
ptg000023l:1-31662945.30916244	chr14	3965110	4164595
ptg000023l:1-31662945.31115731	chr14	3419478	3965107
ptg000034l:1-2552660.2102886	chr13	5341145	5732177
ptg000034l:1-2552660.2493921	chr22	4756012	4811746
ptg000041l:1-36579044.0	chr21	10974308	43493444
ptg000041l:1-36579044.32519157	chr21	8874250	10981776
ptg000041l:1-36579044.34743316	chr13	12004300	11911689
ptg000050l:1-2649560.0	chr22	4726319	5872794
ptg000050l:1-2649560.94034	chr22	4497857	4726318
ptg000051l:1-95769069.0	chr14	12624545	101435482
ptg000051l:1-95769069.88825502	chr14	11781332	11963118
ptg000051l:1-95769069.89007289	chr14	11400175	11777238
ptg000051l:1-95769069.89139042	chr14	4448799	11402427
ptg000051l:1-95769069.94824504	chr15	3814704	4438381
ptg000051l:1-95769069.95448182	chr14	3492199	3621056
ptg000055l:1-11434566.2203892	chr21	8146412	8874251
ptg000055l:1-11434566.3096637	chr13	12283321	20621247
ptg000085l:1-1069922.0	chr19	20040	42270
ptg000085l:1-1069922.22234	chr9	147007499	147945090
ptg000106l:1-627290.48764	chr16	94798325	95340137

ptg000129c:1-44968.25395	chr14	3392578	3409290
ptg000142l:1-68776.0	chr15	3043282	3063829
ptg000142l:1-68776.25372	chr15	3052332	3083365
ptg000142l:1-68776.56388	chr22	5512086	5524472
ptg000147c:1-90021.38392	chr22	5966074	5975956

Table S2 The misassembly positions in Han1 draft assembly.

Chromosome	Misassembly position on Han1 draft assembly	Type of misassembly
Chr13	459,155	Wrong order misassembly
Chr13	485,169	Wrong order misassembly
Chr14	2,763,805	haplotype variant
Chr14	5,306,367	haplotype variant
Chr15	19,546,254	Wrong order misassembly
Chr22	6,523,416	haplotype variant
Chr22	11,003,500	haplotype variant

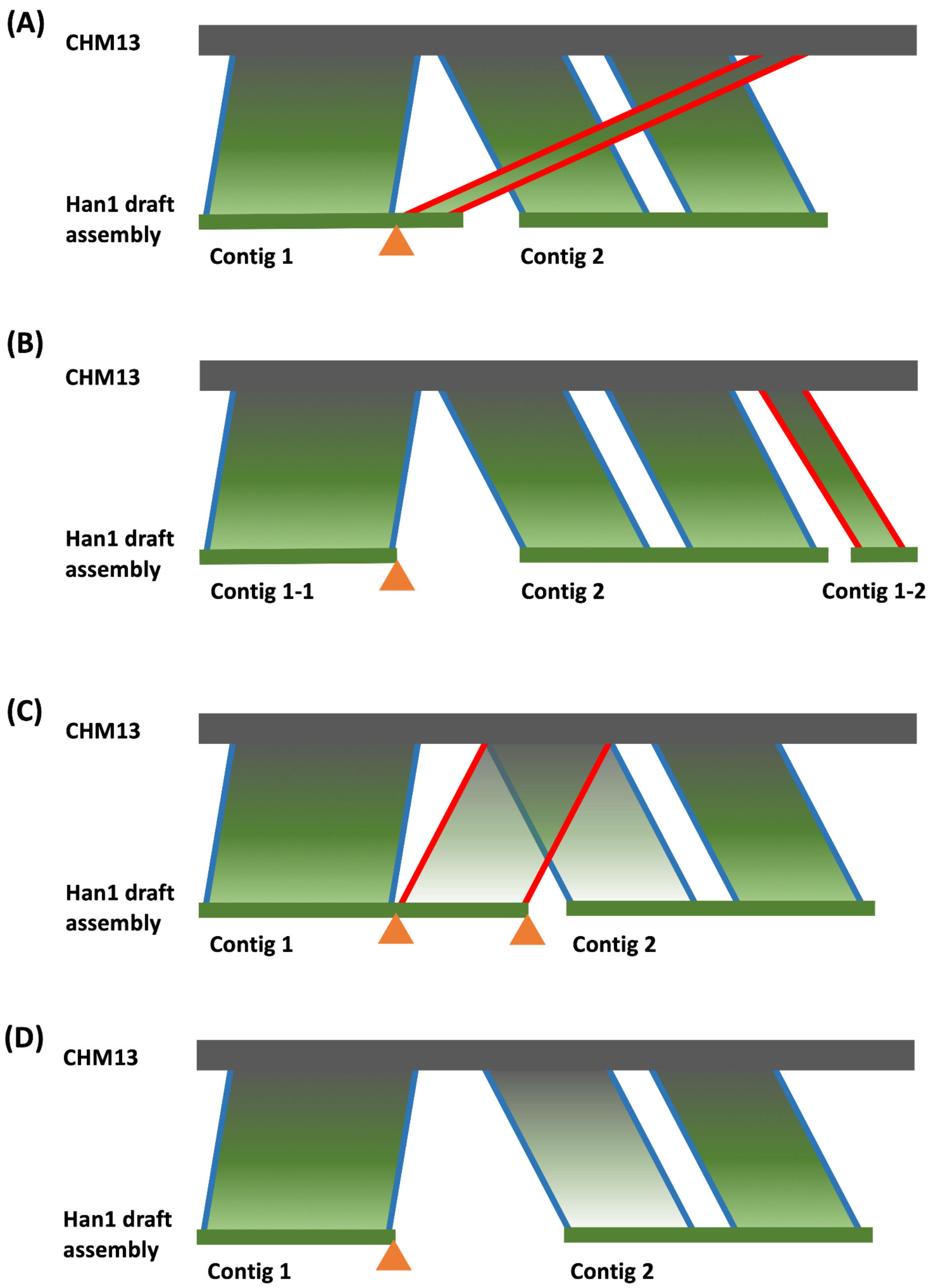

Figure S1 The schematic plots show the process of solving misassembled contigs in Han1 draft assembly. (A) and (B) show the wrong-order misassembly. (A) shows two contigs in Han1 draft assembly, Contig1 and Contig 2, aligning to T2T-CHM13. The parallelograms with blue borders represent alignments between T2T-CHM13 and Han1 draft assembly that are in the correct order whereas the parallelogram with the red border represents the alignment that is in the wrong order. The orange triangle pointed at the missassembly position on the Han1 draft assembly. (B) shows our strategy to fix the misassemblies. We split Contig 1 into Contig 1-1 and Contig 1-2, moved Contig 1-2 to the back so that it is in the same order as T2T-CHM13, and re-ran SAMBA scaffolder to fill in gaps using contigs of ONT Flye assembly or T2T-CHM13 sequences. In total, we detected three wrong-order misassemblies in the Han1 draft assembly. This type of misassembly was found in repetitive regions like centromeres and telomeres.

(C) and (D) show the redundant haplotype variant. (C) shows that both the later part of Contig 1 and the front part of Contig 2 map to the same region on T2T-CHM13. We compared the alignment score for both alignments and removed the one with the lower quality. In this example, the alignment highlighted with the red parallelogram was removed. The two boundaries of this haplotype-variant are marked with orange triangles. (D) We trimmed Contig 1 to the first orange triangle and re-ran SAMBA scaffolder to fill in gaps using contigs of ONT Flye assembly or T2T-CHM13 sequences. We detected 2 redundant haplotype variants on chr14 and chr22 in the Han1 draft assembly and recorded 4 positions. In total, we detected 7 misassembly positions in the Han1 draft assembly after our scaffolding pipeline and listed them in **Table S4**.

Table S3 Genes that have at least one homozygous, non-SNP mutation in Han1 as compared to T2T-CHM13. Genes whose names begin with "LOC" are proteins with no known function. Genes whose names begin with "OR" are olfactory receptors. A frameshift is an insertion or deletion in the coding portion of the transcript that is not a multiple of 3 in length. A 3′ truncation denotes a truncated transcript where the lost sequence will produce a shorter protein. A "start lost" is either a truncation that deletes the 5′ end of the transcript including the start codon, or a mutation that changes the start codon to a different codon. A "stop gain" refers to a mutation that creates a stop codon, producing a shorter protein without necessarily altering the transcript length.

Mutation group	Gene name
frameshift	AQP12A,<br>DEFB126,<br>GOLGA6L10,<br>IGLV4-60,<br>KLHDC7B,<br>LOC105373102,<br>LOC105375947,<br>LOC112268186,<br>LOC124900476,<br>LOC124900994,<br>LOC124900995,<br>LOC124901041,<br>LOC124901234,<br>LOC124903219,<br>LOC124903621,<br>LOC124903828,<br>LOC124903856,<br>LOC124904770,<br>LOC124904774,<br>LOC124908048,<br>MUC19,<br>NBPF19,<br>OR4L1,<br>OR7G3,<br>RP1L1,<br>TMEM82,<br>TRAJ52
3′ truncation	IGKV7-3,<br>LOC124901069,<br>LOC124901481,<br>LOC124904063,<br>LOC124904417,<br>LOC124905956,<br>OR1E2,<br>OR4E1,<br>OR4F29,<br>OR51I2,<br>PBOV1,<br>RETNLB,<br>TCP11X1,<br>TPSB2
start lost	LOC105377805,<br>LOC124903229,<br>LOC124905153
stop gained	KIR2DL3,<br>LOC124901163

Table S4 Genes that have more than one copy fewer in Han1 compared to T2T-CHM13.

Copy number in T2T-CHM13	Genes in T2T-CHM13	Copy number in Han1	Genes in Han1
5	IGHVIII-13-1_4, IGHVIII-13-1_1, IGHVIII-13-1, IGHVIII-13-1_3, IGHVIII-13-1_2	3	IGHVIII-13-1_4, IGHVIII-13-1_1, IGHVIII-13-1_3
7	TBC1D3K,TBC1D3,TBC1D3D, TBC1D3L, TBC1D3G,TBC1D3H,TBC1D3B	4	TBC1D3,TBC1D3L, TBC1D3H,TBC1D3B
9	AMY2A, AMY1A, AMY1B, AMY1C_3, AMY1C_1, AMY1C_4, AMY1C_2, AMY1C, AMY2B	7	AMY2A, AMY1A, AMY1B, AMY1C_3, AMY1C_2, AMY1C, AMY2B
10	SPDYE8, SPDYE12, SPDYE11, SPDYE10, SPDYE14, SPDYE13_1, SPDYE13, SPDYE17, SPDYE9, SPDYE15	7	SPDYE8, SPDYE12, SPDYE11, SPDYE10, SPDYE13_1, SPDYE17, SPDYE9
34	FAM90A14_11, FAM90A16_1, FAM90A14_6, FAM90A14_5, FAM90A14_9, FAM90A14_7, FAM90A10, FAM90A1, FAM90A9_1, FAM90A9, FAM90A19, FAM90A23_4, FAM90A16, FAM90A23, FAM90A9_2, FAM90A23_1, FAM90A16_3, FAM90A22, FAM90A16_2, FAM90A14_1, FAM90A23_3, FAM90A26, FAM90A14_2, FAM90A23_2, FAM90A8, FAM90A7, FAM90A17, FAM90A14, FAM90A14_4, FAM90A14_10, FAM90A18, FAM90A14_8, FAM90A14_3, FAM90A14_12	16	FAM90A14_5, FAM90A14_7, FAM90A1, FAM90A9_1, FAM90A19, FAM90A23_4, FAM90A23, FAM90A23_1, FAM90A22, FAM90A23_3, FAM90A26, FAM90A23_2, FAM90A8, FAM90A7, FAM90A17, FAM90A14_8

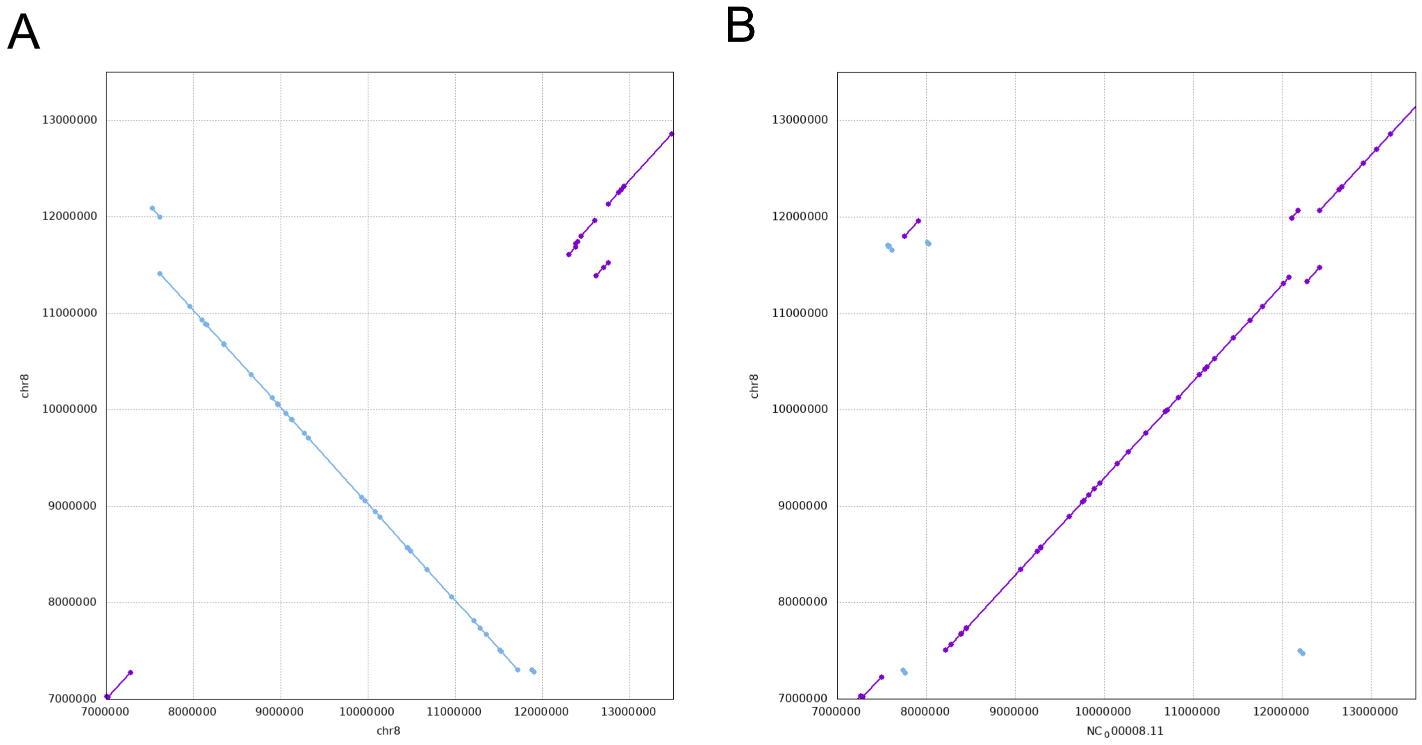

Figure S2 The zoomed-in dotplots on chromosome 8 from 7,000,000 to 13,500,000 showing the complex β-defensin gene cluster locus visualized by mummerplot. The segments in purple color mean sequences in T2T-CHM13 and Han1 are in the same direction whereas the blue color means they are in the reverse direction. (A) demonstrates the inversion between T2T-CHM13 and Han1 in this region with T2T-CHM13 on the X axis and Han1 on the Y axis. (B) shows the collinearity between GRCh38 and Han1 in this region with T2T-CHM13 on the X axis and Han1 on the Y axis.
